# Radiotherapy with 15 × 2.633 Gy vs. 20 × 2.0 Gy in Patients with Malignant Spinal Cord Compression and Favorable Survival Prognoses: A Secondary Analysis of the RAMSES-01 Trial

**DOI:** 10.3390/cancers16203436

**Published:** 2024-10-10

**Authors:** Dirk Rades, Darejan Lomidze, Natalia Jankarashvili, Fernando Lopez Campos, Arturo Navarro-Martin, Barbara Segedin, Blaz Groselj, Christian Staackmann, Nathan Y. Yu, Jon Cacicedo

**Affiliations:** 1 Department of Radiation Oncology, University of Lubeck, 23562 Lubeck, Germany; christian.staackmann@uksh.de; 2 Radiation Oncology Department, Tbilisi State Medical University, Tbilisi 0177, Georgia; dlomidze@hotmail.com; 3 Radiation Oncology Department, Ingorokva High Medical Technology University Clinic, Tbilisi 0177, Georgia; 4 Department of Radiation Oncology, Acad. F. Todua Medical Center-Research Institute of Clinical Medicine, Tbilisi 0112, Georgia; natnataliaj@yahoo.com; 5 Department of Radiation Oncology, University Hospital Ramón y Cajal, 28034 Madrid, Spain; fernando_lopez_campos@hotmail.com; 6 Department of Radiation Oncology, Instituto Catalán de Oncología, 08908 l’Hospitalet de Llobregat, Barcelona, Spain; arturonavarro7@icloud.com; 7 Department of Radiotherapy, Institute of Oncology Ljubljana, 1000 Ljubljana, Slovenia; bsegedin@onko-i.si (B.S.); bgroselj@onko-i.si (B.G.); 8 Department of Radiotherapy, Faculty of Medicine, University of Ljubljana, 1000 Ljubljana, Slovenia; 9 Department of Radiation Oncology, Mayo Clinic, Phoenix, AZ 85054, USA; yu.nathan@mayo.edu; 10 Department of Radiation Oncology, Cruces University Hospital, 48903 Barakaldo, Bizkaia, Spain; jon.cacicedofernandezbobadilla@osakidetza.eus; 11 Department of Radiation Oncology, Biobizkaia Health Research Institute, 48903 Barakaldo, Bizkaia, Spain

**Keywords:** malignant spinal cord compression, radiotherapy alone, favorable survival prognosis, higher doses, overall treatment time, secondary analysis

## Abstract

**Simple Summary:**

This study compared 15 × 2.633 Gy over three weeks to 20 × 2.0 Gy over four weeks for malignant spinal cord compression (MSCC) in patients with favorable survival prognoses not receiving upfront neurosurgery. After a propensity score adjustment of the Cox and logistic regression models, no significant differences were observed regarding local progression-free survival (LPFS), overall survival (OS), and post-treatment ambulatory status. A trend (*p* = 0.073) was found in favor of 15 × 2.633 Gy regarding improvement of motor function. Considering the limitations of our study, 15 × 2.633 Gy may be preferable for patients with MSCC with favorable prognosis.

**Abstract:**

Many patients with malignant spinal cord compression (MSCC) who are not candidates for neurosurgery receive radiotherapy alone. This study compared 15 × 2.633 Gy over three weeks to 20 × 2.0 Gy over four weeks in patients with favorable survival prognoses. The outcomes of 34 patients treated with 15 × 2.633 Gy (equivalent dose 41.6 Gy_10_) in the RAMSES-01 trial were compared to 239 patients from an existing database receiving 20 × 2.0 Gy using propensity-score-adjusted Cox and logistic regression models. All patients had favorable survival prognoses. Endpoints included local progression-free survival (LPFS), improvement of motor function, post-treatment ambulatory status, and overall survival (OS). After propensity score adjustment, the 12-month rates of LPFS and OS were 98.1% (RAMSES-01 cohort) vs. 91.6% (*p* = 0.265) and 79.1% vs. 82.2% (*p* = 0.704), respectively. Regarding improvement of motor function, 15 × 2.633 Gy appeared superior (*p* = 0.073). No significant difference was observed regarding ambulatory status (*p* = 0.822). The three-week regimen for MSCC has similar outcomes and reduces treatment time when compared to a four-week regimen.

## 1. Introduction

Malignant spinal cord compression (MSCC) is not uncommon in patients with solid cancers or hematologic malignancies [1,2]. Real or true MSCC is often defined as a malignant lesion (metastasis from solid cancer, myeloma lesion, or affection by lymphoma) that infiltrates or compresses the spinal cord and leads to neurologic, particularly motor, deficits [2]. If the lesion causes pain without neurologic deficits, the clinical picture may be called impending MSCC. True MSCC is an emergency situation and often requires fast and multidisciplinary decisions regarding the optimal treatment. The vast majority of patients with MSCC receive radiation treatment, either alone or preceded by neurosurgery [1,2]. Upfront neurosurgery was demonstrated to improve the outcomes of selected patients with a comparably good performance status, an estimated survival time of at least three months, without total paraplegia for longer than 48 h, with restriction of MSCC to one spinal area, and with MSCC from a tumor not considered very radiosensitive [3]. Nonetheless, a considerable number of patients, particularly those who do not meet the above criteria, receive radiotherapy alone.

One of the most important factors in determining dose fractionation for MSCC is prognosis. In general, dose-fractionation regimens for MSCC include single-fraction and multi-fraction treatments mostly lasting between two and four weeks [1,2]. The most commonly administered regimens include 1 × 8.0 Gy, 5 × 4.0 Gy (one week), and 10 × 3.0 Gy (two weeks) [2]. In addition, many centers use 20 × 2.0 Gy (four weeks) for patients with longer expected survival. The survival time following a course of radiotherapy can be judged by means of a validated scoring instrument, which considers six patient-related factors, i.e., type of malignancy leading to spinal metastases causing MSCC, the period between the initial diagnosis of the malignancy and MSCC, the presence of additional bone metastases or other non-osseous metastases, pre-treatment walking ability, and the dynamic of the progress of motor deficits [4]. Possible scoring points range between 20 and 46 points. In the original study, 12-month overall survival (OS) rates were 70% in patients achieving 36 to 40 points and 89% in patients achieving more than 40 points [4]. Thus, patients with 36 or more points were defined as patients with favorable survival prognoses.

Patients considered candidates for radiotherapy alone with a poor prognosis should be treated with a single-fraction treatment or a short course (lasting about one week) of radiotherapy [1,2,4,5,6]. However, patients with a high probability of surviving considerably longer, i.e., for at least 12 months following their radiation treatment, may benefit from irradiation with higher total doses. In a previous retrospective study that investigated patients with MSCC and longer estimated survival (at least 36 points when using the above-mentioned scoring tool), 191 patients receiving 10 × 3.0 Gy (two weeks) were matched and compared to 191 patients irradiated with 15 × 2.5 Gy (three weeks) or 20 × 2.0 Gy (four weeks) [7]. The equivalent doses in 2 Gy fractions (EQD2) for tumor cell killing (α/β ratio 10 Gy) were 32.5 Gy_10_ (10 × 3.0 Gy), 39.1 Gy_10_, (15 × 2.5 Gy), and 40.0 Gy_10_ (20 × 2.0 Gy) [7,8,9]. In this study, patients receiving one of the two higher-dose regimens had significantly better local progression-free survival (LPFS) and OS rates at 1 and at 2 years compared to 10 × 3.0 Gy [7].

The results of the matched-pair study provided the impetus for the RAMSES-01 trial published in March 2024 [10,11]. This international multi-center phase II trial (one-arm) investigated radiotherapy alone with 18 × 2.333 Gy or 15 × 2.633 Gy in 50 (of 62 planned) patients with MSCC who achieved 36 or more points when using the above-mentioned scoring instrument [4]. The endpoints of the RAMSES-01 trial included LPFS, OS, improvement of motor and sensory functions, post-radiotherapy ambulatory status, and relief of pain and distress [10,11]. In addition, the patients of the RAMSES-01 trial were compared to a historical control group of 266 patients with MSCC and favorable survival prognoses irradiated with 10 × 3.0 Gy [11]. The patients of the RAMSES-01 group achieved significantly better LPFS (primary endpoint) and showed a strong trend towards improvement of motor function when compared to the control group [11]. Since the previous matched-pair study suggested that 15 × 2.5 Gy and 20 × 2.0 Gy were also superior to 10 × 3.0 Gy, it would be interesting to know how these regimens do perform in comparison to the regimens used in the RAMSES-01 trial [10,11]. The present study compared 15 × 2.633 Gy, one of the RAMSES-01 regimens, to the more common regimen 20 × 2.0 Gy. If these two regimens were similarly effective, 15 × 2.633 Gy would be preferable, since its overall treatment time is shorter. This would mean fewer trips to the radiation oncology department or a shorter hospital stay for the patients.

## 2. Materials and Methods

This international multi-center study compared 34 patients with motor deficits caused by MSCC and longer expected survival who had received radiotherapy without preceding neurosurgery in a previous phase II trial (RAMSES-01) to 239 patients from an existing database who were irradiated with 20 × 2.0 Gy for MSCC with existing motor deficits (Table 1). All of these patients achieved 36 or more points on a validated tool created to predict the patient’s probability to survive following a course of radiotherapy for MSCC [4]. Both cohorts were compared with respect to LPFS, improvement of motor function (within six months following irradiation), post-treatment ambulatory status, and OS by applying propensity score adjusted Cox and logistic regression models. These models considered several factors, including the patient’s age at the time of the first radiation fraction (≤64 years vs. ≥65 years), gender (female vs. male), time period between the first diagnosis of the malignancy until the irradiation of MSCC (≤15 vs. >15 months), bone metastases in addition to those lesions causing MSCC (no vs. yes), hematogenous metastases outside bony structures (no vs. yes), type of the malignancy leading to spinal metastasis and consecutive MSCC (breast cancer vs. prostate cancer vs. multiple myeloma or lymphoma vs. lung cancer vs. less radiosensitive malignancies such as renal cell carcinoma or sarcoma vs. other malignancies), pre-radiotherapy dynamic of the development of motor deficits due to MSCC (faster: 8–14 days vs. slower: >14 days; motor deficits did not develop very fast, i.e., within <8 days, in any patient, i.e., within 1–7 days), pre-treatment ability to walk (no vs. yes: with or without support by crutches or walker), number of metastatic vertebrae associated with the compression of the spinal cord (1 or 2 vs. 3 or more), and performance score according to the Eastern Cooperative Oncology Group (ECOG 0–2 vs. 3–4). Both the original RAMSES-01 trial and the current study were approved by the responsible ethics committee in Lübeck, Germany (AZ 18-360 and 2024-491, respectively).

The phase II part of the RAMSES-01 trial included two dose-fractionation regimens lasting 3 weeks or 3.5 weeks, respectively. The majority of these patients received the 3 weeks regimen, namely 15 × 2.633 Gy. This regimen represented an EQD2 of 41.6 Gy_10_ and a biologically effective dose (BED) of 49.9 Gy_10_ with respect to tumor cell killing (α/β ratio = 10 Gy) [8,9]. The maximum relative total dose at the spinal cord was 101.2%, representing an EQD2 of 46.6 Gy_2_ for the risk of myelopathy (α/β ratio = 2 Gy), which was considered safe [8,9]. One patient considered as an emergency started with one fraction of 3.0 Gy followed by 12 fractions of 2.633 Gy and two fractions of 2.333 Gy. This regimen represented an EQD2 of 41.4 Gy_10_ for tumor cell killing and an EQD2 of 45.5 Gy_2_ for the risk of myelopathy. Prior to the first contact with a radiation oncologist, patients were evaluated by a neurosurgeon for the need for surgical intervention prior to irradiation. MSCC was diagnosed by spinal computed tomography (CT) or spinal magnetic resonance imaging (MRI). The planning target volume included the involved vertebrae plus 1 cm above and below and was covered by the 95% isodose. Radiotherapy generally started the day the patient was presented to the Radiation Oncology department or the following working day. It was recommended that the patients receive concurrent dexamethasone during their course of radiotherapy.

Initially, the outcomes of the 34 patients, who were treated within the RAMSES-01 trial between August 2019 and December 2021, with respect to LPFS, OS, effect of radiotherapy on motor function, post-treatment ambulatory status, partial and complete pain relief, and toxicity (according to the Common Terminology Criteria for Adverse Events) [12], were described. The definition of partial pain relief included improvement by ≥2 points without an increase in opioid analgesics and/or a decrease in opioid analgesics by 25% or more without increased pain [13]. Distress was assessed using the National Comprehensive Cancer Network Distress Thermometer, ranging between 0 points (no distress) and 10 points (maximum distress) [14]. The relief of distress was defined as a decrease by ≥2 points when compare to the pre-radiotherapy scores.

Subsequently, these patients were retrospectively compared to a historical control group from an anonymized multi-center database. The patients of the control group had motor deficits due to MSCC and favorable survival prognoses (≥36 points on a validated survival score [4]). They were treated with 20 × 2.0 Gy (BED = 48.0 Gy_10_ for tumor cell killing, EQD2 = 40.0 Gy for both tumor cell killing and risk of myelopathy) of conventional radiotherapy between 1998 and 2011. In the majority of these patients, treatment volumes encompassed one healthy vertebra above and below the metastatic lesions. The baseline characteristics without a propensity score adjustment of the RAMSES-01 group and the control group are shown in Table 1. The main endpoint of this comparative study was LPFS at 12 months. LPFS was defined as an improvement or no further deterioration of motor deficits during the course of radiotherapy and freedom from an in-field recurrence of MSCC (confirmed by MRI or CT) with motor deficits after the radiation treatment. If patients experienced an out-field recurrence of MSCC (outside the irradiated parts of the spine), they were censored for the analysis of LPFS. The further investigated endpoints were improvement of motor function by radiotherapy (maximum improvement during follow-up), post-treatment walking ability, and OS. Motor function was graded as follows: 0 = no motor deficits, 1 = motor deficits, walking ability without support (e.g., cutches, walker), 2 = motor deficits, walking ability requiring support (e.g., walker or crutches), 3 = inability to walk, 4 = complete paraplegia) [15]. Improvement represented a decrease by at least 1 point and deterioration an increase by at least 1 point, respectively [15].

### Statistical Considerations

LPFS, and OS were calculated from the last day of the radiation treatment. Since the follow-up period in the RAMSES-01 trial was 12 months, the maximum follow-up of the control group was also set to 12 months. The procedure was performed to decrease the risk of a bias due to different lengths of follow-up. The comparisons between the patients treated with 15 × 2.633 Gy in the RAMSES-01 trial, and the historical control group treated with 20 × 2.0 Gy with respect to LPFS and OS, were performed using Cox regression models with independent variables for treatment and the logit of the propensity scores. The pre-specified model allowed for a non-linear association between propensity scores and outcome link function by the so-called ‘one-spline’ approach [16]. A restricted cubic spline transformation was applied consisting of cubic functions between knots and linear functions in the tails. Five knots were positioned at the percentiles (equally spaced) of the log odds. The comparisons regarding improvement of motor function following radiotherapy and post-treatment ambulatory status were performed with logistic regression models using the same propensity adjustment methods as described above. For the statistical analyses, the SAS software (version 9.4; SAS, Cary, NC, USA) was used. These models were chosen instead of other frequently applied approaches including matching the patients one to one and the inverse probability of weighting the treatment, since the numbers of events (local progression, death) were expected to be low.

## 3. Results

### 3.1. Outcomes in the RAMSES-01 Group

The 34 patients who were irradiated in the RAMSES-01 phase II trial with 15 fractions of radiotherapy were included in the presents study. These patients received highly conformal irradiation with volumetric modulated arc therapy (VMAT, 31 patients) or intensity-modulated radiation therapy (IMRT, three patients). For all patients, concurrent dexamethasone was recommended. However, 16 patients did not agree to the concurrent medication, were identified to have contraindications, or had very minor motor deficits. The other 18 patients received a median dose of 8 mg per day (range 4–16 mg). In the patients treated in the RAMSES-01 trial, the 12-month rates of LPFS and OS were 97.1% and 67.6%, respectively. Following their radiation treatment, 20 patients (58.8%) experienced an improvement of motor deficits. In the other 14 patients (41.2%), further progression of motor deficits was stopped. Thus, the overall response rate was 100%. The post-treatment ambulatory rate was 94.1% (32 of 34 patients). Of the 33 patients with vertebral pain prior to radiotherapy, 28 patients (84.8%) achieved at least partial and six patients (18.2%) complete pain relief, respectively. Moreover, 29 patients (85.3%) reported relief of distress [14]. Acute radiation-related toxicity was absent or mild in 31 patients (91.2%). Grade 2 toxicity (nausea) occurred in two patients (5.9%) and grade 3 toxicity (diarrhea) in one additional patient (2.9%). Late radiation-related toxicity was not observed.

### 3.2. Comparison of the RAMSES-01 Group and the Historical Control Group

**Figure 1:**
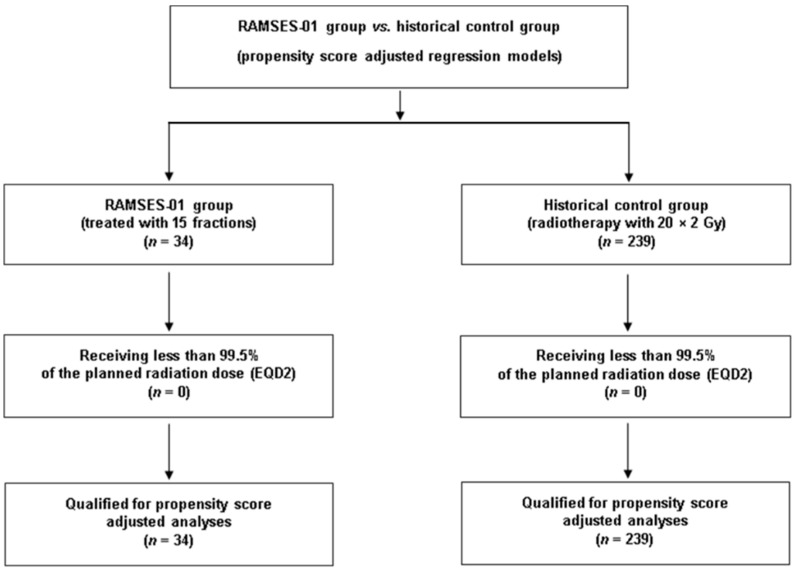
A CONSORT diagram for the comparison of the RAMSES-01 group and the control group.

**Figure 2:**
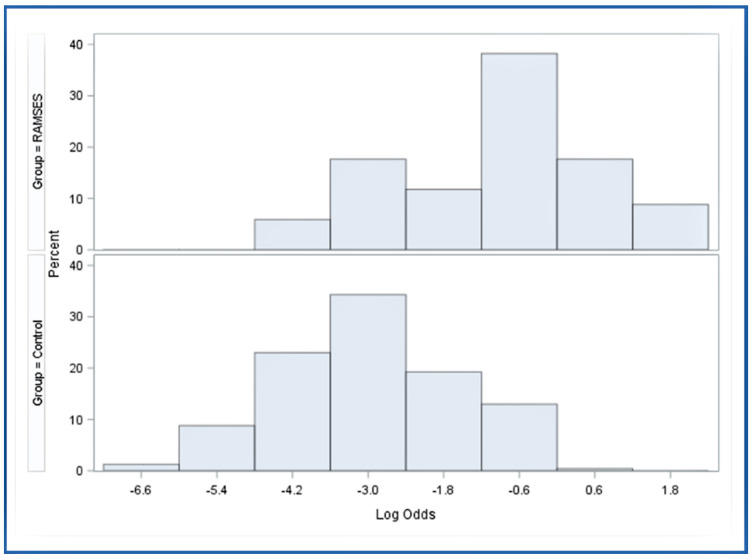
The distribution of the propensity scores stratified by patient cohort on the logit scale (*p* < 0.001, Wilcoxon two-sample test).

Two-hundred-thirty-nine patients met the criteria for the control group, including a favorable survival prognosis and reception of at least 99.5% of the planned EQD2 (Figure 1). The patients of the control group received concurrent treatment with corticosteroids (daily doses of 4–32 mg dexamethasone). Figure 2 illustrates the significant heterogeneity of the distribution of the propensity scores between the RAMSES-01 group and the control group, demonstrating the necessity for adjustment.

**Figure 3:**
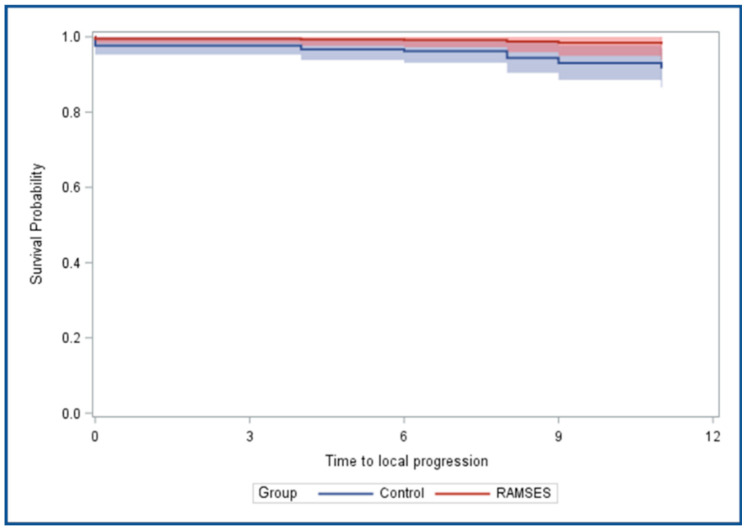

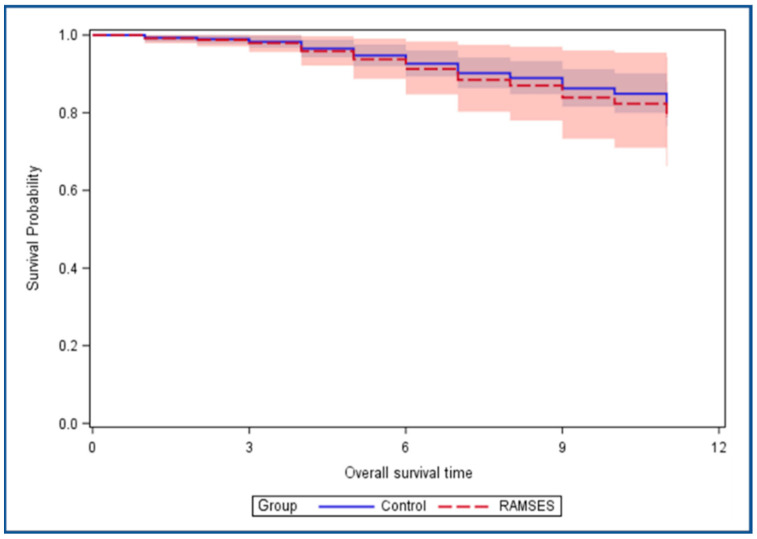
Estimated local progression-free survival (LPFS, **top**) and overall survival (OS, **bottom**) after propensity score adjustment. The adjusted LPFS and OS curves are shown, plus the 95% confidence limits (average of predicted LPFS and OS for all group-specific patients based on a propensity-score-adjusted proportional hazards model).

After propensity score adjustment using a Cox regression model, the 12-month LPFS rates were 98.1% (95% confidence interval: 93.9–100%) in the RAMSES-01 group and 91.6% (86.8–96.9%) in the control group, respectively (*p* = 0.265) (Figure 3). The 12-month OS rates were 79.1% (66.2–94.4%) and 82.0% (76.6–87.9%), respectively (*p* = 0.704) (Figure 3). The results of the comparisons of both groups with respect to LPFS and OS (Cox regression model with propensity score adjustment) are summarized in Table 2. Moreover, after propensity score adjustment using a logistic regression model, a trend was found in favor of the RAMSES-01 group regarding improvement of motor function (*p* = 0.073), whereas no significant difference was observed regarding post-treatment ambulatory status (*p* = 0.822). The results of these comparisons are also given in Table 2.

## 4. Discussion

Despite the increasing use of upfront neurosurgical intervention, many patients presenting with motor deficits due to MSCC still receive radiotherapy alone [1,2,3,17]. It is generally agreed that patients with very poor or poor expected survival should receive single-fraction treatment or a short course of multi-fraction treatment, whereas for patients expected to survive longer, multi-fraction treatment with higher total doses (≥30 Gy) and longer overall treatment times (two to four weeks) appear more appropriate, since they result in improved LPFS when compared to single-fraction or short-course radiotherapy [1,2,4,5,6,18,19,20]. The frequently used dose-fractionation regimens of longer-course treatment include 10 × 3.0 Gy, 15 × 2.5 Gy, and 20 × 2.0 Gy [1,2,18,20].

Studies directly comparing longer-course radiotherapy regimens in patients with MSCC and favorable survival prognoses are rare. In 2011, a retrospective study compared 15 × 2.5 Gy or 20 × 2.0 Gy to 10 × 3.0 Gy in a cohort of 382 patients with MSCC and a high probability to survive for 12 months or longer following radiotherapy alone [7]. The patients of both groups were matched with respect to age, gender, performance status, type of malignancy, number of vertebrae with MSCC, other osseous or visceral metastases, the interval between the first diagnosis of the malignancy to MSCC, walking ability, and the dynamic of motor deficits. Radiotherapy with higher doses was associated with significantly better long-term LPFS and OS [7]. Unfortunately, both higher-dose regimens were combined to one group, and subgroup analyses separately considering or comparing 15 × 2.5 Gy and 20 × 2.0 Gy were not performed. Recently, two alternative longer-course regimens, namely 15 × 2.633 Gy over three weeks and 18 × 2.333 Gy over three-and-a-half weeks, were recently investigated in the RAMSES-01 phase II trial [11]. These regimens were compared to 10 × 3.0 Gy (historical control group) over two weeks and appeared superior with respect to LPFS and improvement of motor function. This could be explained by the higher EQD2 regarding the tumor cell killing of the regimens of the RAMSES-01 trial [8,9,10,11].

Since both the higher-dose regimens of the retrospective matched-pair study and the regimens of the RAMSES-01 trial were suggested to result in better outcomes than 10 × 3.0 Gy, a study comparing the regimens of both studies would be desirable [7,10,11]. In this study, we compared 15 × 2.633 Gy and 20 × 2.0 Gy. Initially, we looked specifically at the group of 34 patients treated with 15 fractions in the RAMSES-01 trial, which has not been assessed before. These patients had 12-month LPFS and OS rates of 97.1% and 67.6%, respectively, which were similar to those found in the entire cohort of the RAMSES-01 trial, including patients treated with 18 fractions (96.8% and 69.9%, respectively) [11]. Moreover, in the 34 patients receiving 15 fractions who were included in the present study, the rates regarding improvement of motor deficits and post-treatment walking ability were 58.8% and 94.1%, respectively, which were also similar to the entire cohort of the RAMSES-01 trial (56.0% and 94.0%, respectively) [11]. The overall and complete relief of pain were achieved by 84.8% and 18.2% of patients in the present study, compared to 84.4% and 22.2% in the entire cohort of the RAMSES-01 trial [11]. Moreover, the rate of pain relief of 84.8% in the present study was higher than in previous prospective trials (58–81%) in patients receiving radiotherapy alone for any painful bone metastases [21,22,23,24,25]. Grade ≥ 2 and grade 3 toxicity rates were lower in the present study (8.8% and 2.9%) than in the entire cohort of the RAMSES-01 trial (24.0% and 4.0%) [11]. Thus, 15 × 2.633 Gy may be preferable to 18 × 2.333 Gy.

In the main part of this study, 15 × 2.633 Gy was compared to 20 × 2.0 Gy. After propensity score adjusted Cox and logistic regression models, a trend was found in favor of 15 × 2.633 Gy with respect to improvement of motor function. This is a very important endpoint in the treatment of MSCC. Otherwise, treatment outcomes were not significantly different between both treatment groups. Moreover, the outcomes of the current 15 × 2.633 Gy group were better than those found in patients receiving 15 × 2.5 Gy or 20 × 2 Gy in the previous matched-pair study [7]. Considering these results and the shorter overall treatment time of 15 × 2.633 Gy, this regimen appears preferable to 20 × 2.0 Gy in patients with MSCC and longer expected survival who are not suitable for or do not wish to receive upfront neurosurgery.

### Limitations of the Study

Although 15 × 2.633 Gy was shown to be at least as effective as 20 × 2 Gy with respect to improvement of motor deficits and LPFS, one has to be aware of the limitations of our present study. These include the retrospective nature of the control group and the different time periods during which the patients were irradiated. Since the patients of the control group were irradiated prior to 2012, they were not treated using VMAT or IMRT. Moreover, the treatment volumes were different between both groups. However, since the subject of this comparative study is metastatic disease and one major objective is symptom palliation, other radiotherapy techniques such as Three-Dimensional Conformal Radiation Therapy (3D-CRT) can also be considered effective for MSCC. In general, more sophisticated techniques like VMAT and IMRT may require more preparation time than 3D-CRT. The main reason why VMAT and IMRT were used in the RAMSES-01 trial is the higher precision of the radiation treatment, which allowed us to limit the maximum dose at the spinal cord to 101.2% of the prescribed dose. This was considered important in order to keep the risk of radiation myelopathy very low, particularly in patients with a favorable survival prognosis. The risk of myelopathy increases with lifetime.

Despite propensity score adjustment with the use of Cox regression and logistic regression models considering ten characteristics, including primary tumor type, a certain risk of hidden selection biases remains. Particularly, since the Bilsky score was not available for the historical control group and, therefore, patients could not be matched for this score, the two cohorts may not be adequately balanced in this regard. This would have had an impact on our results, since higher Bilsky scores were suggested to be associated with a worse post-treatment modified McCormick scale score in a previous retrospective study [26]. However, 90-day readmissions, the need for re-operation, long-term local control, and time to local recurrence were not significantly different between patients with Bilsky 0–1 and patients with Bilsky 2–3 lesions [26].

The results of our study may not be valid for patients receiving upfront neurosurgery, a treatment that is increasingly used for patients with MSCC and comparably good survival prognoses [27,28,29,30,31]. Neurosurgical approaches include the comparably novel technique of separation surgery, which may be followed by focal radiotherapy such as stereotactic body radiation therapy (SBRT) [28,29,30,31]. SBRT alone, which is widely used to treat spinal metastases without MSCC, may also be an option for selected patients with a very limited number of lesions associated with MSCC [32,33,34]. However, one has to keep in mind that further escalation of the radiation dose may be associated with an increased risk of vertebral compression fractures, particularly after single-fraction SBRT [32,35,36]. Another option for MSCC requiring further investigation is neoadjuvant radiotherapy followed by neurosurgery [37]. Moreover, future studies of patients with MSCC and favorable survival prognoses may include periods of follow-up longer than 12 months. Since the follow-up in the RAMSES-01 trial was limited to 12 months, it could not be further extended in the present study. Since our study has some limitations, it should be considered mainly hypothesis-generating.

## 5. Conclusions

Radiotherapy with 15 × 2.633 Gy over three weeks appeared as effective as 20 × 2.0 Gy over four weeks with respect to LPFS. Moreover, a trend was found favoring 15 × 2.633 Gy regarding improvement of motor function, which is a clinically important endpoint. Since 15 × 2.633 Gy was well tolerated, the benefit of the reduction in the overall treatment time was not impaired by treatment-related toxicity. Considering the limitations of the current comparative study, 15 × 2.633 Gy appears preferable for patients with MSCC and favorable survival prognoses assigned to radiotherapy alone, since this regimen means a reduction in the treatment time by one week when compared to 20 × 2.0 Gy. However, these results cannot be generalized to patients with poor or intermediate survival prognoses and to patients with favorable prognoses receiving upfront neurosurgery. Additional clinical trials are needed to better define the optimal dose-fractionation regimens for definitive and post-operative radiotherapy in patients with favorable survival prognoses. These may include trials that investigate focal radiotherapy techniques and a further increase in the radiation dose. Moreover, these trials should focus on specific tumor types or include a number of patients sufficiently large to allow for stratification by primary tumor type.

## Figures and Tables

**Table 1:** Characteristics of the patients treated within the RAMSES-01 trial (n = 34) and the control group treated with 20 × 2.0 Gy (n = 239) without propensity score adjustment.

Characteristic	RAMSES Group N Patients (%)	Control Group N Patients (%)
**Age**		
≤64 years	15 (44.1)	136 (56.9)
≥65 years (elderly)	19 (55.9)	103 (43.1)
**Gender**		
Female	10 (29.4)	120 (50.2)
Male	24 (70.6)	119 (49.8)
**Interval diagnosis of malignancy to MSCC**		
≤15 months	14 (41.2)	82 (34.3)
>15 months	20 (58.8)	158 (65.7)
**Additional bone metastases**		
No	8 (23.5)	132 (55.2)
Yes	26 (76.5)	107 (44.8)
**Hematogenous metastases outside bone**		
No	29 (85.3)	227 (95.0)
Yes	5 (14.7)	12 (5.0)
**Type of malignancy**		
Breast cancer	6 (17.6)	80 (33.5)
Prostate cancer	14 (41.2)	42 (17.6)
Myeloma/lymphoma	7 (20.6)	70 (29.3)
Lung cancer	4 (11.8)	11 (4.6)
Less radiosensitive tumors	2 (5.9)	8 (3.3)
Other malignancies	1 (2.9)	28 (11.7)
**Dynamic (time developing) of motor deficits**		
8–14 days	7 (20.6)	59 (24.7)
>14 days	27 (79.4)	180 (75.3)
**Pre-treatment walking ability**		
Not able to walk	4 (11.8)	22 (9.2)
Able to walk	30 (88.2)	217 (90.8)
**Number of vertebrae associated with MSCC**		
1 or 2	20 (58.8)	122 (51.0)
3 or more	14 (41.2)	117 (49.0)
**ECOG performance score**		
0–2	22 (64.7)	197 (82.4)
3–4	12 (35.3)	42 (17.6)

ECOG: Eastern Cooperative Oncology Group; MSCC: malignant spinal cord compression.

**Table 2:** A comparison of the RAMSES-01 group and the historical control group regarding local progression-free survival and overall survival (Cox regression model with propensity score adjustment) and regarding improvement of motor function and post-treatment walking ability (logistic regression model with propensity score adjustment).

Endpoint	Hazard Ratio Estimate	95% Confidence Interval	*p*-Value
**Local progression-free survival**			
Propensity score adjusted	0.211	0.014–3.254	0.265
**Overall survival**			
Propensity score adjusted	1.195	0.477–2.994	0.704
**Endpoint**	**Odds Ratio Estimate**	**95% Confidence Interval**	***p*-Value**
**Improvement of motor function**			
Propensity score adjusted	2.236	0.929–5.382	0.073
**Post-treatment walking ability**			
Propensity score adjusted	0.800	0.114–5.591	0.822

## Data Availability

Data of the RAMSES-01 trial are available at clinicaltrials.gov (NCT04043156). Other data analyzed for this study cannot be shared (data protection regulations).
